# Development and Evaluation of Graphene Oxide-Enhanced Chitosan Sponges as a Potential Antimicrobial Wound Dressing for Infected Wound Management

**DOI:** 10.3390/ijms26157403

**Published:** 2025-07-31

**Authors:** Przemysław Sareło, Maria Wiśniewska-Wrona, Monika Sikora, Bartosz Mielan, Yuriy Gerasymchuk, Anna Wędzyńska, Vitalii Boiko, Dariusz Hreniak, Maria Szymonowicz, Beata Sobieszczańska, Magdalena Wawrzyńska

**Affiliations:** 1 Pre-Clinical Research Center, Wrocław Medical University, Karola Marcinkowskiego 1, 50-368 Wrocław, Poland; b.mielan@umw.edu.pl (B.M.); maria.szymonowicz@umw.edu.pl (M.S.); magdalena.wawrzynska@umw.edu.pl (M.W.); 2 Department of Biomedical Engineering, Faculty of Fundamental Problems of Technology, Wrocław University of Science and Technology, Wybrzeże Stanisława Wyspiańskiego 27, 50-370 Wrocław, Poland; 3 Centre for the Circular Economy, ŁUKASIEWICZ Research Network—Łódź Institute of Technology, Marii Skłodowskiej-Curie 19/27, 90-570 Łódź, Poland; maria.wisniewska-wrona@lit.lukasiewicz.gov.pl (M.W.-W.); monika.sikora@lit.lukasiewicz.gov.pl (M.S.); 4 Division of Optical Spectroscopy, Institute of Low Temperature and Structure Research, Polish Academy of Sciences, Okólna 2, 50-422 Wrocław, Poland; y.gerasymchuk@intibs.pl (Y.G.); a.wedzynska@intibs.pl (A.W.); v.boiko@intibs.pl (V.B.); d.hreniak@intibs.pl (D.H.); 5 Department of Microbiology, Faculty of Medicine, Wrocław Medical University, Tytusa Chałubińskiego 4, 50-368 Wrocław, Poland; beata.sobieszczanska@umw.edu.pl

**Keywords:** graphene oxide, chitosan, dressings, infected chronic wound, biocompatibility, antimicrobial activity

## Abstract

Chronic infected wounds remain a major medical challenge, particularly in the context of increasing antibiotic resistance. The objective of this study was to develop and evaluate chitosan-based (CS) sponges enhanced with graphene oxide (GO) as potential antimicrobial wound dressings. The composite sponges were fabricated using microcrystalline CS (MKCh) and 5% (*w*/*w*) GO, followed by freeze-drying and γ-sterilization (25 kGy). Physico-mechanical characterization showed that GO incorporation did not significantly alter tensile strength, while absorption and sorption capacities were improved, especially after sterilization. Structural and spectroscopic analyses confirmed increased porosity and molecular interaction between CS and GO. Cytocompatibility was verified in vitro using L-929 fibroblasts, with no cytotoxic effects observed in indirect contact. Antimicrobial activity tests demonstrated that GO-modified dressings exhibited enhanced activity against *E. coli* and *S. aureus*, though results were strain-dependent and not uniformly superior to CS alone. Notably, antifungal efficacy against *C. albicans* was reduced with GO addition. Overall, the developed GO-enriched CS sponges present favorable biocompatibility, mechanical resilience, and selective antimicrobial activity, supporting their potential application in chronic wound management. Further optimization of GO concentration and formulation is warranted to maximize antimicrobial efficacy across a broader spectrum of pathogens.

## 1. Introduction

Nowadays, there is an urgent need for new innovative solutions in the field of biomedicine since non-healing wounds infected with multidrug-resistant bacteria remain a major clinical issue and are a significant burden to the medical system [1]. Their incidence is as much as 1–2% of the population, which is similar to that of patients with heart failure and, consequently, constitutes a serious economic problem [2]. Bacterial infection is the most common preventable challenge to wound healing [3]. Moreover, the inadequate management of infected wounds is the primary cause of limb amputation or life-threatening sepsis. Recent progress has been dedicated to adding antimicrobial agents to wound dressing. Although antibiotics have significantly improved options for treating infections, the expanding resistance of microorganisms to antibiotics constitutes a substantial problem worldwide [4]. In the long term, it is linked with an increased amount of their usage in clinical practice, which in turn should be used responsibly [5]. The replacement of existing antimicrobials with novel and efficient alternatives is the immediate demand to alleviate this problem. Thus, an ideal wound dressing should be highly biocompatible, with good mechanical properties and chemical structure, also showing a dynamic role in the wound healing process and able to prevent bacterial infections.

Recent studies on graphene and its derivatives have shown that it is a promising material for biomedical applications and wound dressing [6]. Moreover, several studies also showed that graphene oxide (referred to further as GO), an oxidized form of graphene, exhibits some antibacterial properties [7]. Its antibacterial mechanism is due to both physical and chemical perturbations. The sharp edges of GO flakes contribute to the mechanical degradation of bacterial cell membranes, thereby causing their destruction [8]. The oxygen accumulated on the surface of the GO molecule may have a destructive effect on pathogenic microorganisms. Due to its electronegativity, GO can easily interact with cationic polymers. Nevertheless, GO-based materials tend to aggregate due to strong interplane interactions, which reduce their potential surface area and limit modes of action. Therefore, to avoid aggregation and enhance the antibacterial activity, various functionalization and surface modifications have been performed on GO [9,10,11]. To address this issue, attempts are being made to create composite materials based on natural polymers and GO, the latter as a filler, which are intended to improve the mechanical, functional, and biological properties as well as the final application efficiency of future dressings.

One of the natural polymers that has shown desirable properties from the point of view of dressings is chitosan (referred to further as CS). First of all, it is biocompatible, biodegradable, and characterized by low immunogenicity. The biological activity of CS is determined, among other things, by the susceptibility of this polymer to enzymatic degradation in the presence of lysozyme (an enzyme found in human body fluids), which results in the formation of bioactive oligomers of glucosamine and N-acetyl-D-glucosamine. Previous studies have shown that hexamines, which include N-acetyl-D-glucosamine, accelerate wound granulation and thus accelerate the healing process [12]. Moreover, CS is a biopolymer with bio-stimulating properties, accelerating the influx of phagocytic cells to the wound site, which cleanses the wound of necrotic tissue and stimulates the migration and proliferation of endothelial cells and fibroblasts. Additionally, this polymer also impairs the growth of bacteria and fungi, which reduces the risk of wound infection. The use of CS in the construction of the dressing results from its specific biological properties, which are beneficial in the treatment of difficult-to-heal wounds [13].

The conducted research aimed to develop and produce a new composite biomaterial based on CS with the participation of GO and to assess its physico-mechanical and biological properties, including demonstrating antimicrobial properties. A form of microcrystalline (MKCh) CS was used to construct the bio-composite dressing material, which, in addition to its unique biological properties, is very easily subject to enzymatic degradation within the wound (in the presence of lysozyme) and has a bio-stimulating effect on the reconstruction of damaged tissue. GO was incorporated into the composite to enhance its antimicrobial activity due to its known bacteriostatic and/or bactericidal properties.

## 2. Results

### 2.1. Studies of Physico-Mechanical Parameters of Sponges GO-Enhanced Confirm Its Proper Properties to Be Applied as a Dressing

#### 2.1.1. Determination of Mechanical Parameters of Dressing GO-Enhanced Sponges

For the obtained dressing materials (CS sponge of MKCh) with and without the participation of GO, the assessment of physico-mechanical parameters was conducted. The preparations were tested before and after radiation sterilization (25 kGy). The test results of basic mechanical parameters are presented in Table 1.

Based on the results presented in Table 1, it can be concluded that sterilization with γ rays at a dose of 25 kGy does not affect the values of strength parameters of the produced sponges. However, for sponges with GO, a significant reduction/deterioration of their elasticity in relation to the material before sterilization was observed.

#### 2.1.2. Studies of the Absorption and Sorption Properties of Biocomposite Dressing Materials in the Form of a Sponge

For the obtained dressing materials (CS sponge of MKCh) with and without the participation of GO, the assessment of absorption (i.e., WRV), and sorption properties capacity (i.e., Ws) of 1 g dressing sponges was carried out. The preparations were tested before and after radiation sterilization (25 kGy). The test results are presented below in Table 2 and Table 3.

The test results presented in Table 2 and Table 3 allow us to state that the sorption capacity of both types of sponges (sponges with and without GO) increased during the storage of the preparations in a water bath. For the sponge with GO, an increase in the WRV was observed in comparison with the value of this parameter for the sponge itself and after the sterilization process. It should also be noted that the increase in the sorption and absorption capacity of sponges made of MKCh is significantly influenced by γ radiation, which probably disrupts the structure of the material. Good sorption and absorption properties of the sponges produced result from their porous and developed internal structure, which was confirmed by scanning electron microscopy (SEM, to compare further).

#### 2.1.3. Assessment of the Internal Structure of Dressing Materials in the Form of a Sponge

**Figure 1:**
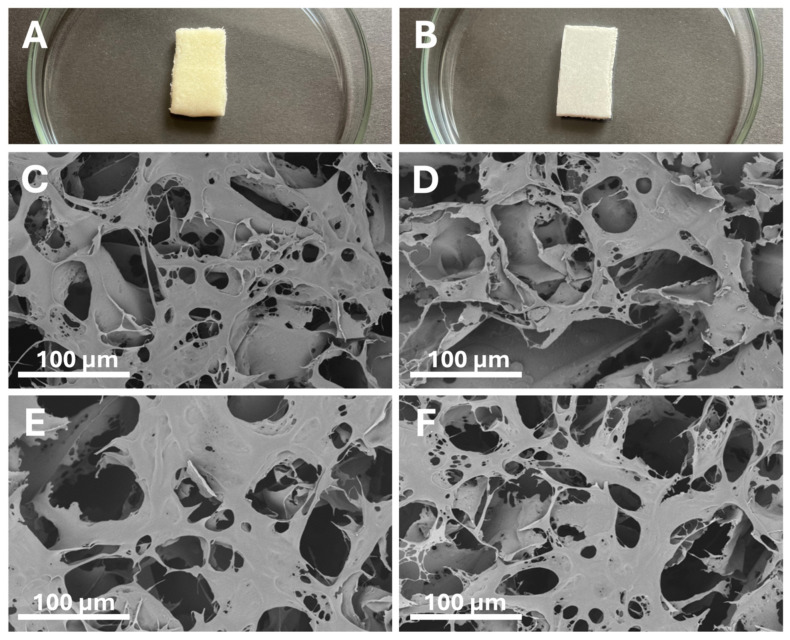
The representative photographic images of the (**A**) sponges made of MKCh and (**B**) sponge made of MKCh with the addition of GO. SEM image of the outer surface of the sponge made of MKCh with and without the addition of GO, where (**C**) MKCh sponge surface before sterilization, (**D**) MKCh + GO sponge surface before sterilization, (**E**) MKCh sponge surface after sterilization (25 kGy), (**F**) MKCh + GO sponge surface after sterilization (25 kGy). Magnification 1500×, presented scale bar indicates 100 μm.

For the obtained dressing materials (CS sponge of MKCh) with and without the participation of GO, the internal structure was assessed using SEM with 1500× magnification. The preparations were tested before and after radiation sterilization (25 kGy)—Figure 1.

SEM photos confirm that the produced MKCh sponges have a well-developed internal surface, characterized by numerous pores of various sizes. The material made of CS in microcrystalline form allows for obtaining high cross-linking of the internal structure, which allows for achieving high sorption capacities by the MKCh sponge. Referring to the research results presented in the article and observations made using a SEM (Figure 1), the reason for the increase in the sorption and absorption capacity of sponges should be sought in the fact that irradiation with γ rays at a dose of 25 kGy causes changes in the internal structure of sponges, resulting in increased porosity and, consequently, an increase in the water absorption capacity.

#### 2.1.4. FTIR Spectroscopy Studies of Potential Molecular Interaction Between CS and GO

**Figure 2:**
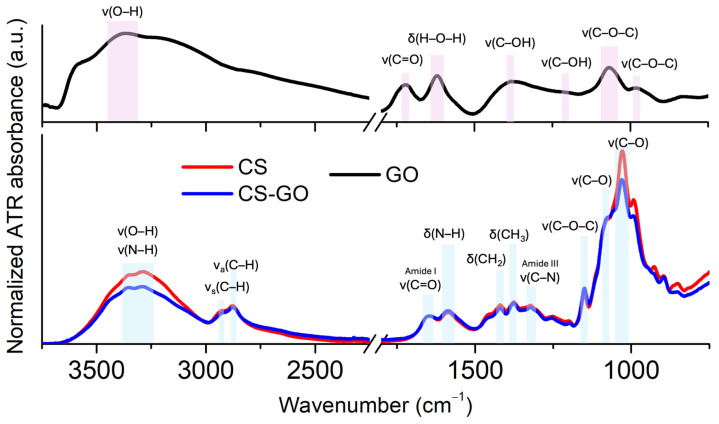
FTIR spectra of GO, CS, and CS–GO composite. The characteristic absorption bands are assigned as follows: for GO—broad O–H stretching (~3360 cm^−1^), C=O stretching (~1720 cm^−1^), H-O–H bending from adsorbed water (~1620 cm^−1^), C–OH stretching (~1380 cm^−1^), C–O (epoxy or phenolic) stretching (~1220 cm^−1^), and C–O–C stretching (~1070 and ~980 cm^−1^); for CS—O–H and N–H stretching (~3350–3280 cm^−1^), C–H stretching (~2922 and ~2868 cm^−1^), Amide I (C=O, ~1647 cm^−1^), N–H bending (~1583 cm^−1^), CH_2_ and CH_3_ deformations (~1417 and ~1375 cm^−1^), C–N stretching (Amide III, ~1325 cm^−1^), C–O–C asymmetric stretching (~1153 cm^−1^), and C–O stretching (~1080 and ~1028 cm^−1^).

GO, CS, and CS-GO composites were characterized by FTIR, and the results are shown in Figure 2.

The FTIR spectrum of GO exhibits several distinct peaks that confirm the presence of oxygen-containing functional groups. The broad peak observed at 3360 cm^−1^ corresponds to the stretching vibrations of hydroxyl (–OH) groups. The band at 1720 cm^−1^ is assigned to the stretching vibration of the carbonyl (C=O) group. The absorption band at 1620 cm^−1^ is currently associated with the bending vibration of adsorbed water molecules (H–O–H bending), but by many authors have so far attributed to vibrations of the C–O–C bonds of epoxy groups [14]. Additional bands include 1380 cm^−1^ that can be attributed to stretching vibrations of C–OH groups, 1220 cm^−1^ that corresponds to C–O stretching in epoxy or phenolic groups, as well as 1070 cm^−1^ and 980 cm^−1^ assigned to C–O–C stretching vibrations, associated with epoxy or alkoxy groups. These observations are consistent with literature data for GO and confirm successful oxidation of graphite and the presence of carboxyl, hydroxyl, epoxy, and other oxygenated functionalities [15,16,17].

The FTIR spectrum of CS displays several characteristic absorption bands confirming its molecular structure. The broad peaks observed at 3350 cm^−1^ and 3280 cm^−1^ are attributed to overlapping O–H and N–H stretching vibrations, including contributions from intramolecular hydrogen bonding. The absorption bands at 2922 cm^−1^ and 2868 cm^−1^ correspond to the asymmetric and symmetric stretching vibrations of C–H bonds, respectively. A strong band around 1647 cm^−1^ is assigned to the C=O stretching vibration of Amide I, while the band at 1583 cm^−1^ arises from N–H bending vibrations of primary amine groups (–NH_2_). The presence of CH_2_ bending and CH_3_ symmetric deformation modes is confirmed by bands at approximately 1417 cm^−1^ and 1375 cm^−1^, respectively. The C–N stretching vibration of Amide III is evident at 1325 cm^−1^. Additionally, the band at 1153 cm^−1^ is attributed to the asymmetric stretching of the C–O–C bridge. Finally, bands at 1080 cm^−1^ and 1028 cm^−1^ correspond to C–O stretching vibrations, further confirming the presence of polysaccharide backbone functionalities [18].

The FTIR spectra of the CS-GO composite show a combination of characteristic peaks of CS and GO. The results implied that interactions existed between CS and GO. The composites of CS-GO are formed due to the reaction between epoxy groups on the GO surface and amino groups (–NH_2_) on the CS surface. Additionally, it has been reported that the formation of CS-GO composite may be due to the formation of a covalent bond after cross-linking between GO and CS, which retards the decomposition of amine units in chitosan [19].

A noteworthy observation for the cross-linked GO composites includes the absence of some bands or changes in intensity when compared to similar FTIR bands for the unmodified GO. For instance, the low molecular weight CS glucopyranose band at 895 cm^−1^ was not observed in GO-CS. The formation of an amide linkage between GO and CS can be demonstrated by the absence of GO peaks at 1720 cm^−1^, attributed to C=O in the −COOH moiety of GO, where a greater FTIR intensity of the band at 1583 cm^−1^ is observed for CS-GO composites. This provides support for the formation of a linkage between GO and CS as the linker, in agreement with previous reports [20].

#### 2.1.5. Raman Spectroscopy in the Characterization of GO in Composite Material

**Figure 3:**
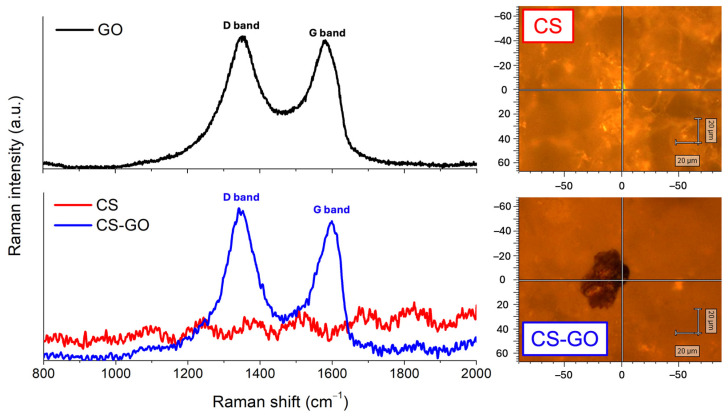
Raman spectra of GO, pure CS-based dressing (without the addition of GO), and CS-GO dressing, respectively. The characteristic D (~1350 cm^−1^) and G (~1580–1600 cm^−1^) bands of GO are clearly visible in both GO and CS-GO samples, confirming the successful incorporation of GO into the dressing matrix. The CS sample does not exhibit these bands. On the right, representative Raman microscope images of the analyzed regions for CS and CS-GO are shown, indicating the precise locations from which the spectra were acquired.

Raman spectroscopy was used to evaluate the structural changes and defect density of GO before and after its incorporation into the CS-based matrix. For GO before introduction to the dressing, characteristic D (~1350 cm^−1^) and G (~1580 cm^−1^) bands were observed (Figure 3). The G band corresponds to the in-plane vibration of sp^2^-hybridized carbon atoms, while the D band is associated with structural defects, disorder, and edge effects in the graphene lattice [21,22].

After incorporation of GO into the CS-based dressing matrix, both D and G bands remained identifiable. Notably, the G band exhibited a shift toward higher wavenumbers, from 1580 cm^−1^ to approximately 1600 cm^−1^ (Figure 3). This blue shift may be attributed to structural changes induced by GO oxidation, an increased number of lattice defects, as well as local strain and electrostatic interactions between the amino groups of CS and the GO surface. Such a shift is commonly reported as evidence of surface functionalization and modification of the local electronic environment of GO [23].

Importantly, the D band did not show a significant increase in intensity, suggesting that the incorporation of GO into the CS matrix did not introduce substantial additional defects or cause significant lattice damage. The observed G band shift strongly indicates successful interaction or functionalization between GO and CS.

### 2.2. Cell Morphology and Viability Studies Proved Cytocompatibility Properties of Dressings

**Figure 4:**
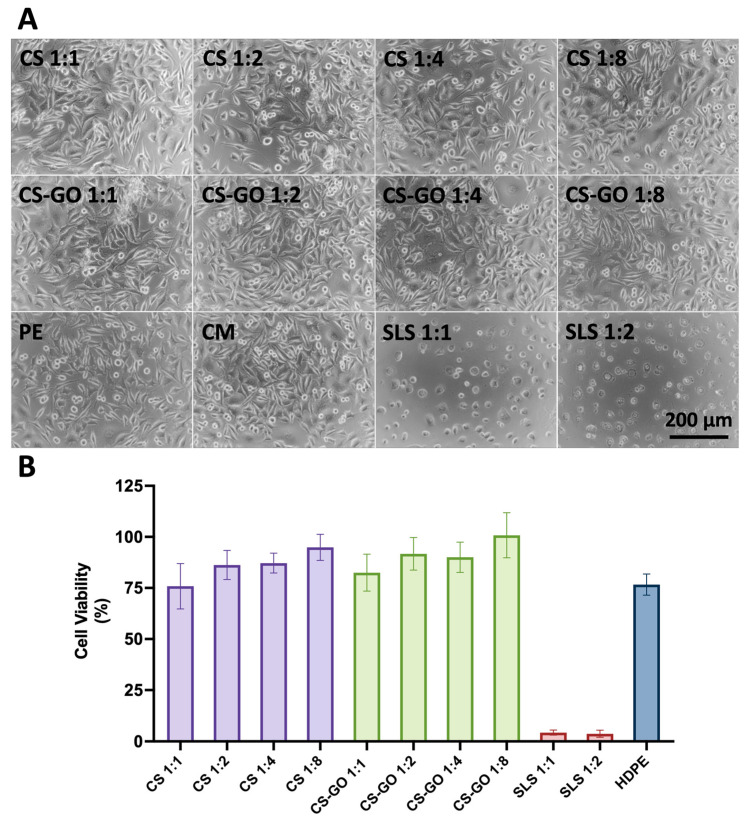
(**A**) Morphology of L-929 cells after 24 h in contact with extracts from sponges made of CS and CS-GO in increasing dilutions (1:1, 1:2, 1:4, 1:8, respectively), as well as morphology of the cells in contact with PE as a negative control, SLS in increasing dilutions (1:1 and 1:2, respectively) as a positive control. Cells cultured in fresh, complete medium served as the reference in this study. Scale bar indicates 200 μm. (**B**) MTT cell viability (mean and standard deviation) expressed as % in comparison to cells cultured in fresh, complete medium (i.e., reference).

As Figure 4 shows, the morphology of L929 fibroblasts in contact with the extract obtained from both samples (CS and CS-GO) is entirely appropriate, and there is no difference between all the dilutions. Additionally, there is no difference in comparison with reference material (i.e., HDPE). Positive controls were performed—cells in contact with SLS in dilutions 1:1 and 1:2 exhibit inappropriate morphology—they are round, the number is significantly lower than in reference materials, and the adhesion is poor. SLS, as expected, has a cytotoxic effect on cells in dilutions 1:1 and 1:2.

MTT test shows clearly that all the extracts of investigated materials (from sponges made of CS in dilutions 1:1, 1:2, 1:4, 1:8, as well as sponges of CS-GO in dilutions 1:1, 1:2, 1:4, 1:8) independently on concentration do not exhibit adverse effect on cells in comparison with reference, cytocompatible material of HDPE. As expected, in positive control (i.e., SLS), cells’ viability is significantly lower.

An important observation is the increased cell viability in comparison to the HDPE control, indicating that the material exhibits properties favorable for cell growth. This feature may be particularly advantageous in the context of the wound healing process.

### 2.3. The Proposed GO-Enhanced CS Dressing Exhibits Potential Anti-Bacterial and Anti-Fungal Activity

**Figure 5:**
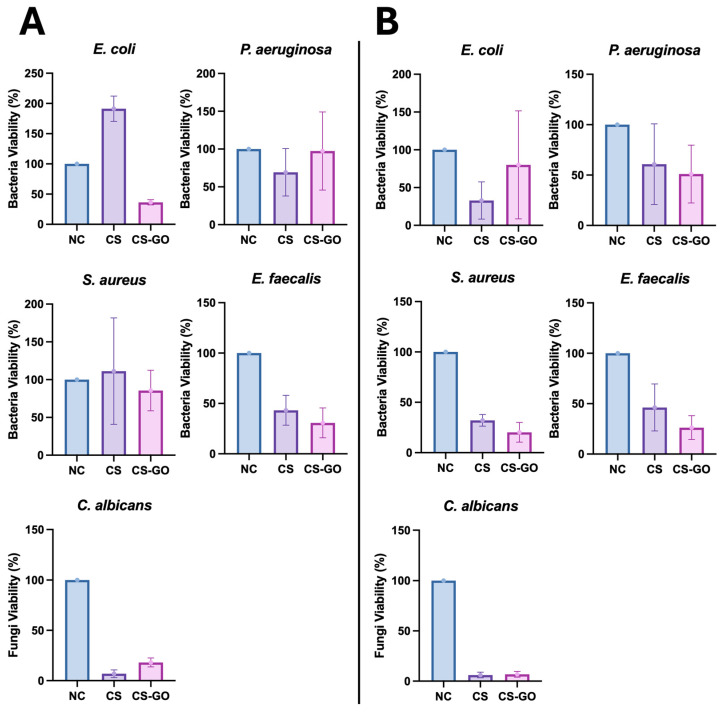
(**A**) The viability (mean and standard deviation) of bacteria and fungi in relation to the negative control (referred to as NC, which was sterile blotting paper) for dressings made of CS and CS enriched with GO (marked as CS-GO). The dressing samples were applied to freshly inoculated tested microorganism species—wound contamination. (**B**) The viability (mean and standard deviation) of bacteria and fungi in relation to the negative control. The dressing samples were applied to inoculated test microorganism species, incubated for 24 h, and then assessed for wound colonization.

The obtained results indicated that the tested dressing model (CS-GO) exhibited better antibacterial activity against freshly inoculated (i.e., wound-contaminating) Gram-negative *E. coli* bacilli (*p* = 0.0005) than those cultured overnight (wound-colonizing). Moreover, the dressing model CS-GO demonstrated antibacterial superiority over CS alone (*p* = 0.01). However, this effect was not observed for another species of Gram-negative bacilli, i.e., *P. aeruginosa*, for which the CS-GO dressing exhibited better bactericidal activity against bacteria cultured overnight (wound-colonizing) on culture medium compared to freshly inoculated bacteria (wound-contaminating) (*p* = 0.009). In the case of *P. aeruginosa*, however, there was no statistically significant difference between CS-GO and CS alone (*p* > 0.05). For Gram-positive cocci of the species *E. faecalis*, the CS-GO dressing exhibited comparable bactericidal activity against wound-contaminating as well as wound-colonizing cocci (*p* > 0.05); however, the addition of GO to the dressing better reduced the viability of enterococci compared to CS alone, although statistically insignificantly. On the other hand, *S. aureus* remained insensitive to CS and CS-GO dressings when applied to freshly inoculated bacteria (i.e., wound-contaminating bacteria) compared to those cultured overnight (*p* = 0.0008). However, when the dressing was applied to bacteria cultured overnight (wound-colonizing) on the culture medium, the CS-GO dressing inhibited their growth more effectively than CS alone (*p* = 0.02). Additionally, CS and CS-GO dressings demonstrated antifungal activity against *C. albicans*, a representative yeast-like fungus that infects chronic wounds after antibiotic treatment, compared to the negative control, i.e., blotting paper. However, supplementation of the CS dressing with GO did not increase its activity against the tested fungal species but even attenuated the activity of CS itself against freshly inoculated *Candida* cells (6.9 ± 4.3% for CS alone vs. 26.5 ± 8% for CS-GO; *p* = 0.0009). However, when applied to overnight culture, both CS and CS-GO demonstrated similar anti-fungal activity (6.1 ± 3.4% vs. 6.8 ± 3.5%; *p* = 0.6). The results from five biological replicates are presented in the graphs below (Figure 5). Nevertheless, CS itself had perfect anti-*Candida albicans* activity.

The bactericidal activity of the CS-GO dressing against microorganisms colonizing wounds (which multiply in the wound) was insufficient for *E. coli* and statistically insignificant for *P. aeruginosa*. The CS dressing without GO supplementation showed better activity against these bacteria. However, the CS-GO dressing was more active against Gram-positive cocci of the *S. aureus* species (*p* = 0.02). In the case of *E. faecalis*, regardless of the presence of GO (*p* > 0.05), CS reduced the number of live bacteria compared to the negative control, i.e., sterile blotting paper. In the case of the bactericidal effect of the tested dressing on *Candida albicans*, the results were very similar to those obtained in the version A experiment. The CS dressing alone reduced the number of live fungal cells very well, but GO did not increase this activity—Figure 5B.

## 3. Discussion

The present study demonstrates the successful development of chitosan (CS)-based wound dressings enhanced with graphene oxide (GO), offering promising multifunctional properties for the management of infected chronic wounds. The combination of microcrystalline chitosan (MKCh) and GO aimed to exploit the inherent biocompatibility and antimicrobial potential of both components, while maintaining mechanical integrity and enhancing fluid-handling capacity—critical features in advanced wound care.

One of the most significant findings of this study is that the incorporation of GO, as well as subsequent sterilization using γ radiation (25 kGy), did not negatively impact the mechanical strength of the CS sponges. While the tensile strength and maximum tensile force remained comparable across all groups, a noticeable reduction in elasticity was observed in the GO-containing sponges after sterilization. This reduction could be attributed to enhanced cross-linking or changes in intermolecular interactions between GO and CS, potentially induced or intensified by γ irradiation [24,25].

Although there are no universally established regulatory standards defining precise mechanical thresholds for wound dressings, several expert recommendations and biomechanical studies suggest minimal functional criteria that dressing materials should meet to ensure safe and effective clinical use. The parameters of the proposed dressing remain within the acceptable range for soft, contact-layer wound dressings. The measured tensile strength of the developed materials, although modest (0.007–0.011 MPa), approaches the recommended threshold of ≥0.01 MPa, which is considered the minimum value required to maintain structural integrity during clinical handling and application. This suggests that the sponges, while soft and compliant, are sufficiently robust for standard dressing manipulation procedures. Additionally, the elongation at maximum tension for all sponge variants ranged from approximately 13% to 20%, which exceeds the minimal functional criterion of ≥ 10–15% necessary for conformability to irregular wound surfaces and movement-related strain. This degree of flexibility is significant in anatomical locations prone to mechanical stress, such as joints or curved body surfaces. Furthermore, the maximum tensile force for all tested samples was within the range of 0.32–0.54 N, thereby meeting the generally accepted threshold of ≥0.3–0.5 N for maintaining dressing structure during application and removal [26,27]. While GO incorporation slightly reduced elasticity in the sterilized variant, the mechanical integrity was preserved. These findings collectively support the suitability of the developed MKCh-GO dressings for clinical use, especially in non-load-bearing or moderately mobile wound environments.

The water retention (WRV) and sorption (Ws) measurements indicated a substantial improvement in fluid-handling properties upon the addition of GO and following sterilization. These enhancements are particularly relevant in the context of chronic wounds, which often produce excessive exudate [28,29]. SEM imaging revealed that the internal porous structure of the sponges became more developed after γ-irradiation, suggesting that radiation-induced microstructural changes facilitated greater water uptake. This outcome aligns with other studies demonstrating that increased porosity correlates with better fluid absorption and oxygen permeability, factors known to support wound healing [30].

FTIR and Raman spectroscopy analyses provided molecular-level confirmation of the successful interaction between CS and GO. FTIR spectra showed the disappearance of key GO peaks and the appearance of new amide-related signals, indicating possible covalent bonding between GO’s oxygen-containing functional groups and the amine groups of CS [19,20]. The Raman shift of the G band to higher wavenumbers following GO incorporation further supports the presence of electrostatic and chemical interactions, reflecting a change in the electronic environment of GO within the composite matrix [21,22,23]. These findings suggest not only the successful dispersion of GO but also stable integration, which is advantageous for long-term functionality and biostability.

Biocompatibility studies using the L-929 fibroblast cell line confirmed that both CS and CS-GO sponges are non-cytotoxic. Moreover, the CS-based dressings exhibited improved cell viability compared to the inert HDPE control. This observation suggests that CS not only provides a non-toxic environment but may also actively support cell proliferation. This effect can be attributed to CS’s structural similarity to glycosaminoglycans, which are naturally present in the extracellular matrix and play a key role in cell adhesion and signaling. Moreover, the enzymatic degradation of CS by lysozyme releases N-acetyl-D-glucosamine oligomers, which are bioactive and may serve as metabolic substrates, thereby further enhancing cell viability and tissue regeneration processes, which are crucial in wound healing [31,32]. This is an essential prerequisite for clinical applications, as dressings are in direct contact with regenerating tissues. Notably, the morphology of cells exposed to extracts from the sponges remained unaltered across all dilutions, indicating that no leachable toxic byproducts were released from the materials. This reaffirms CS’s known cytocompatibility and suggests that GO, at the tested concentration and form, does not introduce cytotoxic effects.

CS, a natural polysaccharide, exhibits broad-spectrum antimicrobial activity against both Gram-positive and Gram-negative bacteria. Its antibacterial effects are primarily based on electrostatic interactions between positively charged amino groups in acidic environments and negatively charged microbial cell walls or membrane components (i.e., lipopolysaccharide, teichoic acids), leading to increased wall and membrane permeability and leakage of intracellular contents. CS may also interfere with microbial DNA transcription or chelate essential metal ions, disrupting bacterial metabolism. These mechanisms support its application in wound dressings designed to reduce the risk of infection [33,34,35]. In addition, GO contributes to antimicrobial activity through both physical and chemical mechanisms. The sharp edges of GO nanosheets can mechanically disrupt bacterial membranes, while oxygen-containing functional groups (e.g., hydroxyl, epoxy, carboxyl) can generate oxidative stress or interact with bacterial surfaces, further compromising membrane integrity. GO via wrapping of bacterial cells by its flakes has also been shown to inhibit the adsorption of nutrients or disturb electron transport processes, inhibiting bacterial growth [6,8,9].

However, it is worth emphasizing that GO does not possess universal antibacterial activity, which largely depends on the physicochemical parameters of GO, such as lateral size, purity, charge, functional groups, degree of oxidation, and hydrophobicity, which result from its preparation technique [36,37,38]. The antibacterial activity of GO is highly dependent on its concentration, which could also have influenced the results of our study. Chaudhary et al. demonstrated that GO at a concentration of 5 g/mL, lower than that used in our study, had little effect on the viability of *E. coli* and *P. aeruginosa*. According to that study, only at a concentration of 80 g/mL did GO exert a bactericidal effect on these species [39]. The antibacterial activity of GO is also influenced by its oxidation state, and GOs with a higher oxygen content exhibit increased antibacterial activity, as they induce oxidative stress in bacteria [40]. On the other hand, bacterial sensitivity to antibacterial substances, including GO, also depends on various factors, such as the growth phase, the presence of surface structures, and the array of antioxidant defenses of the bacterial species to counteract oxidative stress generated by GO [41]. Moreover, environmental factors, such as the presence of other compounds, i.e., zinc or silver ions, or the presence of organic matter, can influence the stability of GO and its antibacterial activity [42,43]. Mokkapati et al. demonstrated that the response of bacteria to GO depends critically on the type of graphene material used (GO vs. its reduced form) and can vary dramatically from one bacterial strain to another, depending on bacterial physiology [44]. Bacteria in the stationary growth phase are more resistant to the antibacterial substances, including GO [45]. Similarly, in our study, when the bacteria were in the logarithmic phase of intensive growth (wound colonization), for most of the tested bacteria (except *E. faecalis*), the tested dressing model showed better activity than in the case of wound contamination, i.e., when the bacteria were exposed to the dressing immediately after inoculation on the medium. In turn, the presence of a layer of exopolysaccharide on *Pseudomonas* spp., a surface structure example, may also modulate the antibacterial activity of GO, for instance, by scavenging oxygen radicals and ions toxic to bacteria. The exopolysaccharide layer is especially abundant on bacterial biofilms and serves as a protective layer. Fallatach et al. demonstrated that exopolysaccharide forms a barrier to protect bacterial cells from physical damage and also acts as a sink for reactive oxygen species produced during oxidative stress, thereby limiting their damaging activity on the cells. GO may also adsorb the components of exopolysaccharide and aggregate, preventing it from coming in contact with bacterial cells [45].

Hence, the results of our study confirmed that GO activity varied against Gram-positive and Gram-negative bacteria, depending on the species and growth phase. While GO-modified sponges demonstrated improved bactericidal activity against *E. coli* and *S. aureus*, the effect was not universal. In fact, in some cases, such as *P. aeruginosa* and *E. faecalis*, the unmodified CS sponge exhibited superior antimicrobial efficacy. This strongly suggests that the composition of CS or the composition of bacterial culture media can influence GO activity. Moreover, GO-modified sponges demonstrated better antibacterial activity against bacterial populations at the log phase of growth than against bacteria that may colonize the wound. We believe that in the case of GO flakes docked in CS, the antibacterial mechanism of the GO flakes’ nano-edges may have been more pronounced than oxidative stress, thereby impacting their activity against different bacterial species. The formation of chemical bonds between GO and CS may reduce the availability of GO’s active edges or oxygen-containing groups, potentially attenuating its antibacterial action. It is also worth noting that while CS alone showed strong antifungal effects against *C. albicans*, the presence of GO reduced this activity. This suggests that GO may interfere with CS’s mode of antifungal action, potentially through alterations to the surface charge or porosity that impact fungal adhesion.

These findings highlight the importance of further optimization. Varying the concentration and particle size of GO, as well as testing different fabrication methods, may allow better tuning of antimicrobial efficacy without compromising cytocompatibility or mechanical performance. Additionally, combining GO with other antimicrobial agents (e.g., silver nanoparticles, essential oils, or peptides) could provide synergistic effects and broaden the antimicrobial spectrum, including activity against resistant strains or biofilms [46].

In summary, the primary objective of this study—to develop and evaluate a novel composite biomaterial based on MKCh with the incorporation of GO—was successfully achieved. The produced sponges demonstrated favorable physico-mechanical properties, including adequate tensile strength and enhanced absorption and sorption capacities, particularly after γ-sterilization. Structural analyses (SEM, FTIR, and Raman spectroscopy) confirmed the uniform porous architecture and molecular integration between CS and GO, indicating the effective formation of the composite. Notably, the materials exhibited excellent cytocompatibility in vitro, with no cytotoxic effects observed at any of the tested dilutions. Moreover, the GO-enriched composites exhibited selective antimicrobial activity, particularly against *E. coli* and *S. aureus*, validating the anticipated enhancement of antimicrobial properties through the incorporation of GO. These findings confirm that the developed MKCh–GO sponge meets the functional and biological requirements of a next-generation wound dressing, thereby fulfilling the original aim of the study.

## 4. Materials and Methods

### 4.1. GO-Enhanced CS Sponges Preparation

#### 4.1.1. CS

In the conducted studies, commercial CS from Primex ehf (Siglufirdi, Iceland) with the following physicochemical parameters was used to produce the polymer formulation: average molecular weight (Mv) = 235.0 kDa, degree of deacetylation (SD) = 86.8%, ash content = 0.60%, secondary swelling index (WRV) = 94.0%.

The suspension of microcrystalline chitosan (MKCh) was prepared by agglomeration from the solution, using a continuous method developed at the Institute in accordance with patent PL 164 247 (Method of producing microcrystalline chitosan using a continuous method) [47]. The developed method enables the production of a product with the assumed molecular and supramolecular structure by adjusting the process conditions. Polymer matrices based on MKCh were produced in the form of a sponge.

#### 4.1.2. GO

The GO was synthesized using a modified version of Brodie’s method, as adapted from the procedure described by Szabo et al. [48]. This involved oxidizing synthetic graphite four times with potassium chlorate (KClO_3_) in fuming nitric acid (HNO_3_) at a temperature range of 60–80 °C, under continuous mechanical stirring for 24 to 48 h The resulting light-yellow suspension was thoroughly washed with deionized water, followed by three rinses with a 10% hydrochloric acid (HCl) solution, and then washed again with water until the pH of the filtrate was neutral (pH = 7.0) [48,49]. The final product was dried in a laboratory oven with forced air circulation at 40 °C for 72 h. The samples were dispersed in water using a high-power ultrasonic homogenizer (20 kHz, 900 W, UZDN-M-900-T, Akademprylad, Ukraine) operated at 70% power for two hours. After sonication, the dispersion appeared homogeneous and stable. To eliminate larger particles, the suspensions were centrifuged twice at 8000 rpm for 10 min.

#### 4.1.3. Production of Composite Materials in the Form of a Sponge with the Participation of GO

These studies aimed to determine the suitability of the developed form of MKCh for producing dressing materials in the form of a sponge. For this purpose, GO was added to the prepared suspension of MKCh with a polymer content of 1.5% by weight in the amount of 5% by weight concerning the polymer content and glycerin in a ratio of 1:0.5. Then, the whole was subjected to thorough homogenization to suspend GO in a paste using a homogenizer type T50 basic (IKA, Warsaw, Poland). The produced composite was subjected to the freeze-drying process using an ALPHA 2-4 freeze-dryer (Martin Christ, Osterode am Harz, Germany). The freeze-drying process was carried out in a temperature range of −22 °C to 15 °C and under a vacuum of 0.1 to 0.06 mbar. Under the given conditions, the total drying time of the preparations ranged from 20 to 24 h, depending on the batch size. The described drying process enabled the production of polymeric materials in the form of a sponge with a uniform, defect-free surface.

The produced sponges were subjected to radiation sterilization at the Institute of Nuclear Chemistry and Technology in Warsaw, at the Radiation Sterilization Station for Medical Devices and Transplants, using a dose of 25 kGy.

### 4.2. Assessment of Physicochemical Parameters of Dressing Sponges Made of MKCh with and Without GO

#### 4.2.1. Determination of Mechanical Parameters of Dressing Sponges

The mechanical parameters of the tested preparations were determined in the Accredited Paper Testing Laboratory Łukasiewicz—ŁIT (accreditation certificate No. AB 388), by the following standards: sponge thickness [mm] according to PN-EN ISO 1923:1999 Porous plastics and rubbers. Determination of linear dimensions [50]; maximum tensile force [N], tensile strength [MPa], and elongation at maximum stress [%], according to PN-EN ISO 1798:2009 Flexible porous plastics. Determination of tensile strength and elongation at break [51].

Before testing, the samples were conditioned for at least 24 h at a temperature of 23 ± 1 °C and a humidity of 50 ± 2%. To determine the above strength parameters, a tensile testing machine, model 5544 (INSTRON, Norwood, MA, USA), was used. The distance between the clamps during the test was L = 10 mm, the clamp travel speed was 10 mm/min, and the width of the tested sample was d = 15 mm. The number of measurements was n = 5.

#### 4.2.2. Studies of the Absorption and Sorption Properties of Dressing Materials in the Form of a Sponge

The study assessed the absorption and sorption properties of the composite biomaterial in sponge form based on the secondary swelling index (WRV) and the sorption index (Ws). The measurements were performed as a function of time from 0.25 h to 20 h.

The absorption properties of biocomposite dressing materials were assessed based on the WRV, which was determined following the standard method according to the relationship (Procedure SPR/BPB/14—Determination of WRV of starting chitosan and microcrystalline chitosan, according to GLP No. G-016, IBWCh (currently Łukasiewicz Research Network—Łódź Institute of Technology), 2005 [52]):

WRV = [(m_1_ − m_0_)/m_0_]·100% (1)

where:

m_1_—sample mass after being kept in water for 20 h and centrifuged (4000 rpm) for 10 min (g);

m_0_—sample mass, previously kept in water for 20 h and centrifuged (4000 rpm) for 10 min after drying at 105 °C.

The sorption capacity of the sponges was determined by gravimetric analysis. 10.0 mL of deionized water was measured into a flat-bottomed container, and an assessed sponge sample measuring 2 × 2 cm and weighing to the nearest 0.0001 g was placed on them. After specified times (0.25 h, 0.5 h, 3.0 h, 5.0 h, and 24.0 h), the samples were removed and weighed. The sorption capacity of the material was expressed as a sorption index, and the amount of absorbed water was calculated per 1 g of biocomposite dressing. The Ws (%) was calculated according to the formula:

Ws = [(M_w_ − M_a_)·100]/M_a_ (2)

where:

M_a_—mass of dry sponge;

M_w_—mass of sponge with water.

#### 4.2.3. Evaluation of the Internal Structure of Dressing Materials in the Form of a Sponge by Means of SEM

The assessment of the sponge structure was conducted using a Quanta 200 SEM (FEI Company, Hillsboro, OR, USA), which is part of the institute’s equipment. The samples were mounted on a jaw table, and then a thin layer of gold (20 nm) was sputtered using a Q150R S vacuum sputter (Quorum Technologies, Ltd., Lewes, UK). After sputtering, the samples were placed in the microscope chamber. The tests were conducted in high-vacuum conditions with an electron beam acceleration voltage of 5 kV, utilizing the analySIS Docu program from Soft Imaging System, which was adapted to operate in the Quanta environment.

#### 4.2.4. Evaluation of the Composite Employing ATR-FTIR

ATR-FTIR spectra were recorded using a Nicolet iN10 MX infrared microscope (Thermo Fisher Scientific, Waltham, MA, USA) equipped with a liquid nitrogen-cooled MCT detector and a MicroTip ATR germanium crystal for contact mode recording. The microscope was continuously purged with dry air. All spectra were collected in the range 4000–675 cm^−1^ with a resolution of 4 cm^−1^, averaging 256 scans from the 100 μm × 100 μm sample area. Directly before measuring the sample, the germanium/air background spectrum was recorded as a reference (512 scans, 4 cm^−1^). Spectra were recorded with the OMNIC Picta Software (ver. 1.3, Thermo Fisher Scientific, Waltham, MA, USA). All spectra were analyzed using OriginPro (ver. 2019, OriginLab Corporation, Northampton, MA, USA).

#### 4.2.5. Evaluation of the Composite Employing Raman Spectroscopy

Raman spectra were recorded in the 1000–1800 cm^−1^ range, with a spectral resolution of 4 cm^−1^, averaging 128 scans, using a Renishaw InVia Raman spectrometer equipped with a confocal DM 2500 Leica optical microscope (Wotton-under-Edge, Great Britain, UK), a thermoelectrically cooled Charge Coupled Device (CCD) as a detector, and an argon laser operating at 514 nm, power 50% (~0.5 mW at sample surface), acquisition time 10 s and ten accumulations. All spectra were analyzed using OriginPro (ver. 2019, OriginLab Corporation, Northampton, MA, USA).

### 4.3. Biocompatibility Studies of Biocomposite Dressing Materials in the Form of a Sponge

#### 4.3.1. Cell Line

The studies were performed on the normal mouse fibroblast line L-929 (NCTC clone 929, American Type Culture Collection ATCC^®^, Manassas, VA, USA), which is one of the reference lines used in the in vitro assessment of cytotoxicity.

L-929 cells were cultured in DMEM medium (Lonza, Basel, Switzerland) high glucose (4.5 g/L) with 1% L-glutamine with addition of 10% fetal bovine serum (FBS, Sigma Aldrich, St. Louis, MO, USA) and 100 μg/mL streptomycin and 100 μg/mL penicillin (Sigma Aldrich, St. Louis, MO, USA). The cells were cultured in the HERA cell CO_2_ Incubator 150i (Thermo Scientific, Waltham, MA, USA), with the required parameters of 5% CO_2_, 37 ± 1 °C, and a constant humidity of the incubation chamber, referred to as standard conditions. After thawing, the cells were passaged twice using Trypsin-EDTA 0.25% (Sigma Aldrich, St. Louis, MO, USA).

For the experiment, L-929 fibroblast cells, after trypsinization, were seeded in a 96-well plate (TPP, Trasadingen, Switzerland) at a density of 10^4^ cells per well in culture medium as described earlier. Then, cells were incubated under standard conditions. After 24 h, the medium was exchanged, and cells were subjected to an indirect contact study.

#### 4.3.2. Indirect Cell Contact Study

For this purpose, the extracts of the CS-GO composites were prepared. Extracts were prepared according to PN-EN ISO 10993-12 Biological evaluation of medical devices—Part 12: Sample preparation and reference materials [53] standard in Falcon-type vials (50 mL) with a ratio of 6 cm^2^ of material per 1 mL of complete medium (DMEM with supplements as described above). An extract of high-density polyethylene reference standard (referred to as HDPE) was prepared as a negative control. Samples were kept in an incubator at 37 °C in 5% CO_2_ atmosphere. After 24 h of contact between the materials and the medium, the extract was collected and distributed in a total volume of 100 μL per well in a 96-well plate, where cells were seeded 24 h earlier at increasing dilutions (1:1, 1:2, 1:4, and 1:8, respectively). Additionally, cells grown in fresh, complete medium (without anything added) are referred to as CM. As a positive control, sodium lauryl sulfate (SLS) solution in DMEM was used (0.2 mg/mL) in two dilutions: 1:1 and 1:2. The cells were incubated for 48 h.

#### 4.3.3. Morphology Assessment of the Cells

Morphological changes of fibroblasts were evaluated in an inverted contrast-phase microscope CKX 41 (Olympus, Tokyo, Japan). Appropriate photographic documentation has been prepared.

#### 4.3.4. Assessment of Cell Survival Utilizing Viability and Proliferation Assays

To determine the percentage of fibroblast survival, an MTT-type colorimetric test was chosen following Annex C PN-EN ISO 10993-5:2009 Biological evaluation of medical devices. Part 5: Cytotoxicity studies [54].

An MTT solution was prepared for the studies, where 50 mg of MTT salt (bromide 3[4,5-dimethyl-2-yl]-2,5-diphenyltetrazole) (Sigma Aldrich, St. Louis, MO, USA) was dissolved in 50 mL of PBS. The solution was then sterilized with a syringe filter.

The culture medium was removed from the wells, and then 100 µL of MTT solution (1 mg/mL) was added. The plate was incubated for 2 h at 37 °C in a 5% CO_2_ atmosphere.

After this time, the MTT solution was removed, and 100 µL of isopropanol (Sigma, Aldrich, St. Louis, MO, USA) was added to each well. After 30 min of shaking (until the formazan is completely dissolved), 100 μL samples were taken from each well into a 96-well plate, which was then placed in an Epoch (BioTek, Winooski, VT, USA) spectrophotometer, and an absorbance reading was performed at a wavelength of 570 nm. The measurement of absorbance was used to compare the survival of the cells.

### 4.4. Antimicrobial Properties Evaluation of Biocomposite Dressing Materials

The studies were conducted on microorganisms most commonly associated with chronic wounds, namely *Escherichia coli*, *Pseudomonas aeruginosa*, *Staphylococcus aureus*, *Enterococcus faecalis*, and *Candida albicans*. The strains tested were routinely cultured on agar culture media dedicated to individual species of microorganisms, i.e., *Mueller-Hinton* agar for bacterial species and *Sabourand* medium for *Candida albicans*. The studies were conducted in two versions: (ver. A) antimicrobial activity of the tested dressing on the growth of bacteria and fungi contaminating the wound—in this version, dressing samples were applied to freshly inoculated tested microorganism species on the proper culture media, (ver. B) antimicrobial activity of the tested dressing on bacteria and fungi already present in the wound, i.e., colonizing the wound—in this version, dressing samples were applied to inoculated tested microorganism species on the proper culture media, incubated earlier for 24 h.

Pieces of CS sponge supplemented with GO (model of dressing for chronic wounds) and a piece of blotting paper as the negative control, all with a surface area of 1 cm × 1 cm were placed on agar culture media with freshly inoculated microorganisms (ver. A) or on media with grown strains of microorganisms (ver. B), so that they adhered tightly to the substrate and had complete contact with microorganisms. The suspension of microorganisms in physiological saline, dispersed evenly onto the culture media, always had an OD of 1 × 10^8^ CFU/mL, determined spectrophotometrically. This approach enables the assessment of how the tested dressing affects an infected wound, where bacteria are at various stages of growth, such as logarithmic or stationary (ver. B mimicking infection of the wound), in contrast to a wound freshly contaminated with bacteria from the skin or environment that are unable (ver. A mimicking the colonization of the wound). After 24 h of incubation at 37° C, the dressing samples and negative control blotting paper pieces were removed from the substrate using sterile tweezers, placed in Eppendorf tubes containing 1 mL of sterile physiological saline, and shaken for 10 min (100 rpm) to release any microorganisms present on the dressing samples. The obtained microbial suspensions were diluted in geometric progression and inoculated onto culture media. After overnight incubation, the number of microbial colonies that had grown was counted. The result was calculated as the percentage of microorganisms grown on the substrates compared to the negative control, i.e., a piece of blotting paper with the same surface area as the test samples. The second negative control, to which the results for CS-GO were related, was CS sponges without added GO.

### 4.5. Statistical Analysis

The statistical analysis of the obtained data sets was performed using GraphPad Prism 10 (GraphPad Software, San Diego, CA, USA). The Shapiro–Wilk test was used to assess normality, and the Levene’s test was used to determine homogeneity of variance. The significance level was set to 0.05. The data with a normal distribution were compared using an unpaired *t*-test; otherwise, the Mann–Whitney test was used with a significance level of 0.05.

## 5. Conclusions

The developed MKCh sponges enhanced with GO fulfill the essential physical and mechanical requirements for application as wound dressing materials. These sponges demonstrate adequate mechanical strength as well as excellent sorption and absorption capacities. Spectroscopic analyses confirmed the successful incorporation of GO into the CS sponge matrix and revealed interactions between the dressing components, indicating effective integration of the material. Biocompatibility studies, conducted in accordance with PN-EN ISO 10993 standards, demonstrated that the proposed GO-modified CS dressings are cytocompatible and safe for biomedical use. Microbiological studies suggest that the addition of GO imparts antibacterial properties, potentially enhancing the dressing’s ability to reduce bacterial viability on contaminated or colonized wound surfaces. In summary, the developed GO-enriched CS sponge meets the criteria for a potential biomaterial suitable for treating difficult-to-heal, infected chronic wounds.

## Figures and Tables

**Table 1:** Basic mechanical parameters of dressing materials in the form of sponges of MKCh with and without the addition of GO before and after the sterilization process (25 kGy).

Sample	Thickness (mm)	Max Tensile Force (N)	Tensile Strength (MPa)	Elongation at Max Tension (%)
Sponge of MKCh	3.05 ± 0.06	0.374 ± 0.046	0.008 ± 0.001	14.7 ± 6.4
Sponge of MKCh/25 kGy	3.34 ± 0.30	0.542 ± 0.062	0.011 ± 0.002	17.3 ± 3.4
Sponge of MKCh + GO	3.01 ± 0.09	0.344 ± 0.009	0.008 ± 0.001	19.9 ± 2.3
Sponge of MKCh + GO/25 kGy	3.12 ± 0.12	0.320 ± 0.020	0.007 ± 0.001	13.4 ± 1.8

**Table 2:** Assessment of the sorption capacity of 1 g dressing sponges with and without the addition of GO before and after the sterilization process (25 kGy) in different measurement times (15, 30, 180, 300 min).

Time (min)		Sponge of MKCh		Sponge of MKCh + GO	
		Ws Index (%)	Sorption Capacity (g)	Ws Index (%)	Sorption Capacity (g)
15	Before 25 kGy	1692.15	16.93	1661.87	16.62
	After 25 kGy	1842.10	18.42	1840.22	18.41
30	Before 25 kGy	1904.10	19.04	1818.97	18.19
	After 25 kGy	2059.36	20.60	2012.62	20.13
180	Before 25 kGy	1806.58	19.93	2011.18	20.11
	After 25 kGy	2144.30	21.46	2105.47	21.06
300	Before 25 kGy	2217.43	22.17	2078.38	20.79
	After 25 kGy	2250.74	22.51	2217.30	22.17

**Table 3:** Assessment of the absorption capacity (i.e., WRV) of dressing sponges with and without the addition of GO before and after the sterilization process (25 kGy).

Sample	WRV (%)
Sponge of MKCh	160.0
Sponge of MKCh/25 kGy	165.0
Sponge of MKCh + GO	195.0
Sponge of MKCh + GO/25 kGy	227.0

## Data Availability

The data that support the findings of this study are available from the corresponding author upon reasonable request.

## Abbreviations

The following abbreviations are used in this manuscript:

CS	Chitosan
FBS	Fetal bovine serum
FTIR	Fourier transform infrared spectroscopy.
GO	Graphene oxide
HDPE	High-density polyethylene
MKCh	Microcrystalline chitosan
Mv	Molecular weight
SD	Degree of deacetylation
SEM	Scanning electron microscopy
SLS	Sodium lauryl sulfate
WRV	Secondary swelling index
Ws	Sorption index
